# Multilocus Sequence Typing of *Enterocytozoon bieneusi* Isolates From Various Mammal and Bird Species and Assessment of Population Structure and Substructure

**DOI:** 10.3389/fmicb.2020.01406

**Published:** 2020-06-26

**Authors:** Xiaohua Liu, Yanchen Wu, Fengkun Yang, Baiyan Gong, Yanyan Jiang, Kexin Zhou, Jianping Cao, Weizhe Zhang, Aiqin Liu, Yujuan Shen

**Affiliations:** ^1^Department of Parasitology, Harbin Medical University, Harbin, China; ^2^National Institute of Parasitic Diseases, Chinese Center for Disease Control and Prevention, Chinese Center for Tropical Diseases Research, WHO Collaborating Centre for Tropical Diseases, National Center for International Research on Tropical Diseases, Ministry of Science and Technology, Key Laboratory of Parasite and Vector Biology, MOH, Shanghai, China

**Keywords:** *Enterocytozoon bieneusi*, multilocus sequence typing, population structure, population substructure, evolution

## Abstract

*Enterocytozoon bieneusi* is one of the most common intestinal pathogens in humans and animals. *E. bieneusi* has been confirmed to be complex microsporidian species. Approximately 500 ITS genotypes of *E. bieneusi* have been defined. With the establishment and application of multilocus sequencing typing and population genetic tools in *E. bieneusi*, the studies on these aspects have been carried out worldwide, but little information is available. To understand genetic variation of mini-/micro-satellites and the population structure and substructure of *E. bieneusi* in northeastern China, 305 *E. bieneusi* DNA specimens composed of 28 ITS genotypes were from 13 mammal species and five bird species in the investigated areas. They were characterized by nested-PCR amplification and sequencing at four mini-/micro-satellite loci (MS1, MS3, MS4, and MS7). At the MS1, MS3, MS4, and MS7 loci, 153 (50.16%), 131 (42.95%), 133 (43.61%), and 128 (41.97%) DNA specimens were amplified and sequenced successfully with 44, 17, 26, and 24 genotypes being identified, respectively. Multilocus genotypes (MLGs) showed a higher genetic diversity than ITS genotypes. 48 MLGs were produced out of 90 ITS-positive DNA specimens based on concatenated sequences of all the five genetic loci including ITS. Linkage disequilibrium (LD) and limited genetic recombination were observed by measuring LD using both multilocus sequences and allelic profile data, indicating an overall clonal population structure of *E. bieneusi* in the investigated areas. These data will aid in the longitudinal tracking of the attribution of source of infection/contamination and in elucidating transmission dynamics, and will provide valuable information for making efficient control strategies to intervene with and prevent occurrence of microsporidiosis caused by *E. bieneusi* among animals and transmission of *E. bieneusi* from animals to humans in the investigated areas. Phylogenetic and network analyses identified three different subgroups, revealing the presence of host-shaped segregation and the absence of geographical segregation in *E. bieneusi* population. Meanwhile, the MLGs from zoonotic ITS genotypes were observed to be basically separated from the MLGs from host-adapted ones. Assessment of substructure will have a reference effect on understanding of zoonotic or interspecies transmission of *E. bieneusi* and evolution direction from zoonotic genotypes to host-adapted genotypes.

## Introducion

*Enterocytozoon bieneusi* is the most common microsporidian species (more than 90%) in reported cases of human microsporidiosis worldwide (Matos et al., 2012). Microsporidiosis caused by *E. bieneusi* is characterized by diarrhea, and the severity of diarrhea is closely related to the health status of the infected hosts. Self-limiting diarrhea usually appears in immunocompetent or healthy individuals while chronic or life-threatening diarrhea often occurs in immunocompromised/immunodeficient individuals, such as acquired immune deficiency syndrome (AIDS) patients, organ transplant recipients, cancer patients, travelers, children, and the elderly (Matos et al., 2012; Li and Xiao, 2019; Li et al., 2019a). In addition to humans, *E. bieneusi* has been detected in a variety of animal species, raising a concern of zoonotic transmission (Santín and Fayer, 2011; Wang et al., 2018b). The findings of the same genotypes of *E. bieneusi* in both humans and animals support the presumption of zoonotic potentials (Li et al., 2019b).

*E. bieneusi* has been confirmed to be a complex species with multiple genotypes. With application of genotyping tools and development of genetic markers related to *E. bieneusi*, a deep understanding has been reached about the host specificity and evolution of *E. bieneusi* (Li et al., 2019b). Based on sequence analysis of the internal transcribed spacer (ITS) region of the ribosomal RNA (rRNA) gene of numerous *E. bieneusi* isolates from humans and animals, to date, approximately 500 ITS genotypes of *E. bieneusi* have been identified, and phylogenetic analysis have revealed 11 different phylogenetic groups (groups 1–11) and an outlier with different degrees of host specificity (Li et al., 2019b). Currently, approximately 40 genotypes in group 1 have been identified in humans and multiple animal species, suggesting a large probability of cross-species transmission risks, therefore, this group is considered to be a zoonotic group (Shen et al., 2020). Some genotypes in group 2 (notably BEB4, BEB6, I, and J) have a broader host range than originally thought, increasing their importance for public health. The other genotypes in groups 3–11 seem to have limited or minimal effects on public health due to their more host-specific adaptation (Li et al., 2019a). However, the single ITS locus (243 bp in length) has limitations in representing the whole genome of *E. bieneusi* (∼6 Mb total length), for it does not uncover the subtle changes among *E. bieneusi* isolates closely genetically related to each other at the ITS locus (Widmer and Akiyoshi, 2010; Feng et al., 2011). Therefore, these observations need to be substantiated by sequence characterization of other genetic markers (Santín and Fayer, 2009). A multilocus sequencing tool for high-resolution typing of *E. bieneusi* has been established and widely used to characterize the population structures of *E. bieneusi* isolates based on length polymorphisms and single nucleotide polymorphisms (SNPs) of three microsatellites (MS1, MS3, and MS7) and one minisatellite (MS4) (Feng et al., 2011). To date, MLST analysis have been performed in at least 167 ITS genotypes of *E. bieneusi* in at least ten countries, with 23 from humans and 151 from animals (Supplementary Table S1). Some MLST data have been used to assess population structures of *E. bieneusi* isolates derived from different hosts: AIDS patients (Li et al., 2012, 2013), non-human primates (NHPs) (Karim et al., 2014), pigs (Wan et al., 2016; Wang et al., 2018a; Li et al., 2019; Zhang et al., 2020), foxes and raccoon dogs (Li et al., 2016b), captive giant pandas (Li et al., 2017), and dairy cattle (Wang et al., 2019). Meanwhile, some studies also explored the issues of *E. bieneusi* population genetics in relation to geographical origins and host origins (Li et al., 2013, 2016b, 2017, 2019; Wan et al., 2016; Wang et al., 2018a, 2019; Zhang et al., 2020).

In China, since the first identification of *E. bieneusi* in 2011 (Zhang et al., 2011), molecular epidemiological studies of *E. bieneusi* have been carried out in humans (prevalence: 0.2–22.5%) and animals (prevalence: 0–100%) in at least eight and 29 provinces, municipalities or autonomous regions, respectively, (Gong et al., 2019; Qiu et al., 2019). In northeastern China, *E. bieneusi* is prevalent in mammal and bird species, with the prevalence high up to 89.5% in asymptomatic pigs (Zhao et al., 2014b). However, only two studies reported multilocus genotypes (MLGs) of *E. bieneusi* isolates from two animal species (Wan et al., 2016; Zhang et al., 2016). To further understand the population structure and substructure of *E. bieneusi* in China, 305 *E. bieneusi* isolates, derived from 18 various animal species (mammals and birds) in northeastern China, were characterized by MLST analysis at four mini-/micro-satellite loci (MS1, MS3, MS4, and MS7). Meanwhile, linkage disequilibrium (LD) estimation and phylogenetic and network analyses were also conducted. These results will provide valuable information for making efficient control strategies to intervene with and prevent occurrence of microsporidiosis caused by *E. bieneusi* among animals and transmission of *E. bieneusi* from animals to humans, and will improve our perception about the population structure and substructure of this pathogen.

## Materials and Methods

### Ethics Statement

This study uses *E. bieneusi* isolates obtained from animals in northeastern China. The Research Ethics Committee and the Animal Ethical Committee of Harbin Medical University did not require the study to be reviewed or approved by an ethics committee because all the specimens analyzed in this study were DNA preparations.

### *E. bieneusi* DNA Specimens

**FIGURE 1:**
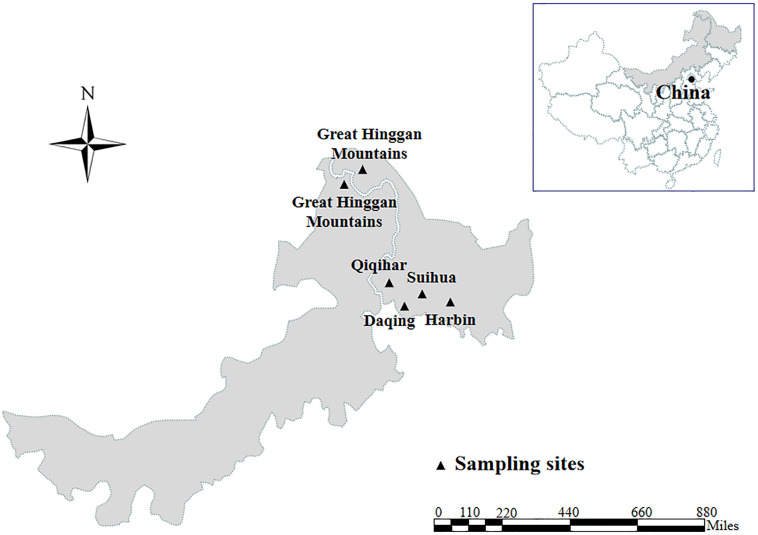
A map showing the sampling sites of DNA specimens of *E. bieneusi* analyzed in this study.

*Enterocytozoon bieneusi* genomic DNA was extracted from each of animal fecal specimens (252 from 13 mammal species and 53 from five bird species) distributing in four cities and Great Hinggan Mountains in northeastern China during the period of May 2012 to June 2019 using a QIAamp DNA Mini Stool Kit (Qiagen, Hilden, Germany) according to the manufacturer recommended procedures (Figure 1). ITS genotypes were identified by nested PCR amplification of an approximately 390 bp nucleotide fragment of the rRNA gene including 243 bp of the ITS region (Buckholt et al., 2002). All the DNA specimens were composed of 28 ITS genotypes belonging to group 1 (*n* = 20), group 2 (*n* = 7) and group 9 (*n* = 1), with some of them having been published in our previous studies (Zhao et al., 2014a,b, 2015a,b,c, 2016, 2017, 2018; Liu et al., 2015; Yang et al., 2016) (Supplementary Table S2). The ITS genotypes, numbers, host origins and phylogenetic groups of all isolates used in the present MLST analysis were showed in Supplementary Table S3.

### Multilocus Sequence Typing

Except for the ITS region of the rRNA gene, all the 305 *E. bieneusi* DNA specimens were also analyzed at the four MLST loci, including three microsatellites (MS1, MS3, and MS7) and one minisatellite (MS4). The primers and the cycle parameters for nested PCR amplifications used in the present study were designed previously by Feng et al. (2011) and the approximately expected fragment lengths of the secondary PCR products were 676 for MS1, 537 for MS3, 885 for MS4 and 471 for MS7 (Feng et al., 2011). TaKaRa Taq DNA polymerase (TaKaRa Bio Inc., Tokyo, Japan) was used in all the PCR reactions. All secondary PCR products were separated by electrophoresis in a 1.5% agarose gel and visualized on GelDoc^TM^ EZ Imager (Bio-Rad, United States) by staining the gel with GelStrain (TransGen Biotech., Beijing, China) before sequencing.

### DNA Sequence Analysis

All secondary PCR products of expected size were directly sequenced with their respective PCR primers on an ABI PRISM^TM^ 3730 DNA Analyzer by Sinogeno-max Biotechnology Co., Ltd. (Beijing, China), using a BigDye Terminator v3.1 Cycle Sequencing kit (Applied Biosystems, Foster City, CA, United States). Two-directional sequencing method was performed to make sure of sequence accuracy. Nucleotide sequences obtained in the present study were compared to all *E. bieneusi* homologous sequences published in GenBank using BLAST searches^1^. They were then aligned and analyzed with each other and reference sequences downloaded from the GenBank database using the program Clustal X 1.83^2^ to determine genotypes at the ITS locus and the genotypes at the MS1, MS3, MS4, and MS7 loci, respectively.

### Analysis of Population Structure

The standardized index of association (*I*^S^_A_) proposed by Habould and Hudson was used to assess the multilocus LD over the allelic profile data using software LIAN 3.5^3^. *I*^S^_A_ equal to zero or with a negative value indicates randomly mating populations and alleles in linkage equilibrium (LE). In contrast, when an *I*^S^_A_ value is greater than zero, there is the existence of non-panmictic population structure and LD in allelic profile data analysis. In addition, the population structure was also assessed by calculating the variance of pairwise differences (*V*_D_) in another test and the 95% critical value (*L*) for *V*_D_. When the *V*_D_ is less than *L*, the population is panmictic and is in LE; otherwise, the population is non-panmictic and a certain degree of LD exists (Haubold et al., 1998). Based on the fact of a very high degree of genetic polymorphism in the ITS region of the rRNA gene within *E. bieneusi*, to assess the influence of the ITS gene on the result of LD analysis, multilocus LD analyses were assessed over the allelic data including and excluding the ITS gene. Gene diversity (Hd), intragenic LD and recombination events (Rms) were also calculated from the concatenated sequences including and excluding the ITS gene without consideration of short insertions and deletions (INDELs) by using DnaSP 5.10.01^4^. The population structure of *E. bieneusi* isolates was assessed by measuring the intragenic LD, *I*^S^_A_, and Rms.

### Analysis of Population Substructure

Phylogenetic relationship of MLGs of *E. bieneusi* was inferred by constructing a neighbor-joining tree of concatenated nucleotide sequences of the five loci (ITS, MS1, MS3, MS4, and MS7) using the program Mega 5^5^ based on the evolutionary distances calculated by Kimura 2-parameter model. The reliability of cluster formation was assessed by the bootstrap analysis with 1,000 replicates. Median-joining analysis implemented in the software network version 4.6.1.0^6^ was conducted with the default parameters to identify a substructure of the *E. bieneusi* isolates.

## Results

### Genetic Polymorphism and MLGs

At the four micro-/mini-satellite loci (MS1, MS3, MS4, and MS7), 153 (50.16%), 131 (42.95%), 133 (43.61%), and 128 (41.97%) DNA specimens were amplified and sequenced successfully, respectively. 44, 17, 26, and 24 genotypes were identified at the MS1, MS3, MS4, and MS7 loci, respectively. SNPs and repeats could be observed: trinucleotide repeats of TGC (*n* = 1), TAA (*n* = 5–38) and T(C)AC(T) (*n* = 3–10) at the MS1 locus, dinucleotide repeats of TA (*n* = 4–17) at the MS3 locus, tetranucleotide repeats of G(A)G(A)TA (*n* = 1–2) at the MS4 locus, and trinucleotide repeats of TAA (*n* = 3–11) at the MS7 locus. Additionally, INDELs were observed at the MS4 locus (Table 1). The nucleotide sequences of all the genotypes were deposited in the GenBank under following accession numbers: MT267413–MT267456 (MS1), MT267457–MT267473 (MS3), MT267363–MT267388 (MS4) and MT267389–MT267412 (MS7).

**TABLE 1:** Amplification efficiency and genetic polymorphism of *E. bieneusi* isolates (*n* = 305) at the MS1, MS3, MS4, and MS7 loci.

Locus	No. of positives (amplification efficiency)	No. of genotypes	Genetic polymorphism	
			Repeat (*n*)	No. of SNPs
MS1	153 (50.16%)	44	TGC (1); TAA (5–38); T(C)AC(T) (3–10)	14
MS3	131 (42.95%)	17	TA (4–17)	36
MS4^a^	133 (43.61%)	26	G(A)G(A)TA (1–2)	39
MS7	128 (41.97%)	24	TAA (3–11)	24

*^a^Insertions and deletions (INDELs) were observed at the MS4 locus.*

In the present study, a total of 90 ITS-positive DNA specimens were amplified and sequenced successfully at the MS1, MS3, MS4, and MS7 loci, producing 48 MLGs based on concatenated sequences of all the five genetic loci including ITS (Supplementary Table S4).

### LD and Population Structure

All the 90 DNA specimens genotyped successfully at all the five genetic loci including ITS were used in LD analysis. Multilocus LD was assessed by the calculation of *I*^S^_A_ based on the allelic profile data of the five genetic loci including ITS using software LIAN 3.5. The value of *I*^S^_A_ (0.5735) was positive and the *V*_D_ (2.5154) was more than *L* (0.8363). A Monte Carlo analysis led to the generation of a significant *P*_MC_ value (<0.01). These above results showed the population of *E. bieneusi* in the investigated areas was in LD with clonality. To exclude the possibility that LD could be attributable to a clonal expansion of one or more MLGs, which could mask the underlying equilibrium, the same MLG was scored as one individual and multilocus LD was assessed again. LD was still existed based on the fact that the *I*^S^_A_ value was still above zero (*I*^S^_A_ = 0.3647), and *V*_D_ (1.1821) was more than *L* (0.5234), suggesting a clonal population structure. As ITS locus has a higher degree of genetic diversity than other genetic loci in *E. bieneusi* genome, the analysis was conducted on allelic profile data excluding ITS. Similar results were obtained, indicating ITS sequences had little influence on the result of LD analysis (Table 2).

**TABLE 2:** Multilocus linkage disequilibrium analysis based on allelic data of *E. bieneusi*.

Population group	No.	*H*	*I*^S^_A_	*P*_MC_	*V*_D_	*L*	*V*_D_ > *L*	LD or LE
ITS included	90	0.8032 ± 0.0366	0.5735	<1.00 × 10^–02^	2.5154	0.8363	Yes	LD
ITS excluded	90	0.8203 ± 0.0417	0.5129	<1.00 × 10^–02^	1.4436	0.6009	Yes	LD
Subgroup 1	52	0.5306 ± 0.1008	0.4432	<1.00 × 10^–02^	2.8890	1.2536	Yes	LD
Subgroup 2	31	0.6935 ± 0.0518	0.4705	<1.00 × 10^–02^	2.9080	1.1424	Yes	LD
Subgroup 3	7	0.7714 ± 0.0614	0.3177	<1.00 × 10^–02^	1.8307	1.2381	Yes	LD
ITS included^a^	48	0.8898 ± 0.0218	0.3647	<1.00 × 10^–02^	1.1821	0.5234	Yes	LD
ITS excluded^a^	46	0.9026 ± 0.0240	0.2695	<1.00 × 10^–02^	0.6232	0.3732	Yes	LD
Subgroup 1^a^	23	0.7043 ± 0.0780	0.2788	<1.00 × 10^–02^	1.9450	1.1813	Yes	LD
Subgroup 2^a^	18	0.7614 ± 0.0693	0.2435	<1.00 × 10^–02^	1.6037	1.0272	Yes	LD

*No identical MLGs were found in subgroup 3. ^a^Considering each group of specimens with the same MLG as one individual.*

The software DnaSP5.10.01 was used to investigate the genetic diversity of *E. bieneusi* isolates. 118 and 90 polymorphic/segregating sites were observed in the concatenated sequence data of five genetic loci including ITS (2584 bp in length) and four genetic loci excluding ITS (2341 bp in length), respectively. LD was also estimated using the *Z_*n*_S* statistics. However, LD was incomplete based on the |D′| *Y* = 0.9750 – 0.0085X (including ITS) and the |D′| *Y* = 0.9857 – 0.0279X (excluding ITS). A negative slope implied a decline in LD with increasing nucleotide distance and the potential occurrence of recombination. Indeed, limited recombination events were detected in analysis of concatenated multilocus sequences (Table 3).

**TABLE 3:** Genetic diversity in *E. bieneusi* isolates (*n* = 90) based on analysis of concatenated multilocus sequences.

Concatenated sequence	No. of site (bp)	No. of Polymorphic (segregating) sites	No. of haplotypes, H	Gene diversity, Hd	Interlocus genetic association (*ZnS*)	95% CI (*ZnS*)	Intragenic linkage disequilibrium (LD) |D′|	Recombination events (Rms)
ITS included	2584	118	41	0.942	0.2217	0.05117–0.37500	*Y* = 0.9750 – 0.0085X	14
ITS excluded	2341	90	39	0.940	0.1766	0.04976–0.34890	*Y* = 0.9857 – 0.0279X	12

*|D′|: linkage disequilibrium (LD), where Y is the LD value, and X is the nucleotide distance in kb.*

### Population Substructure

**FIGURE 2:**
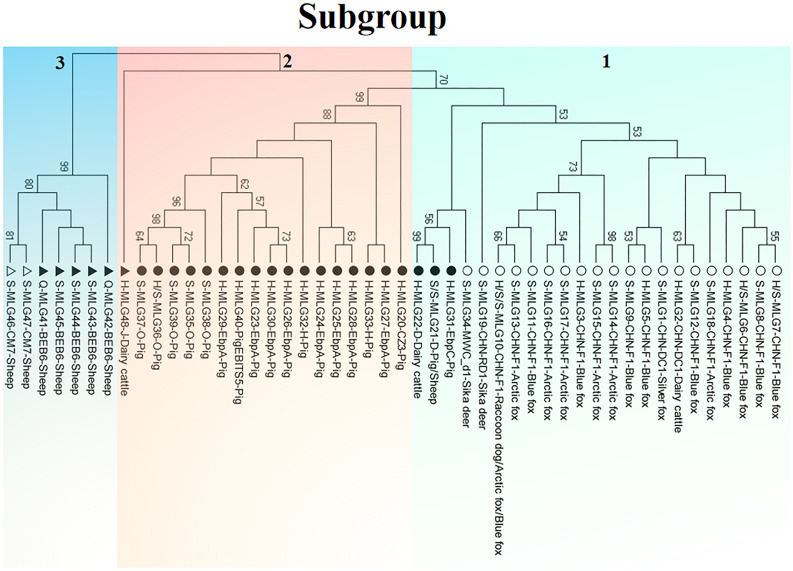
Phylogenetic relationship of multilocus genotypes (MLGs) of animal-derived *E. bieneusi* isolates. The relationship was inferred by a neighbor-joining analysis of 48 concatenated nucleotide sequences of the five genetic loci (ITS, MS1, MS3, MS4, MS7) from 90 *E. bieneusi* isolates belonging to 14 ITS genotypes (CHN-DC1, CHN-F1, CHN-RD1, CZ3, D, EbpA, CHN-RD1, CZ3, D, EbpA, PigEBITS5, BEB6, CM7, and J) from dairy cattle, pigs, sheep, sika deer, blue foxes, arctic foxes, a silver fox, and a raccoon dog (Supplementary Table S4) based on genetic distance by the Kimura 2-parameter model. Each sequence is identified by its sampling site (H: Harbin; Q: Qiqihar; S: Suihua), MLG designation and ITS genotype as well as host origin. The solid and open circles/triangles indicate zoonotic and host-adapted ITS genotypes, respectively, with circle and triangle ones belonging to ITS group 1 and ITS group 2, respectively.

**FIGURE 3:**
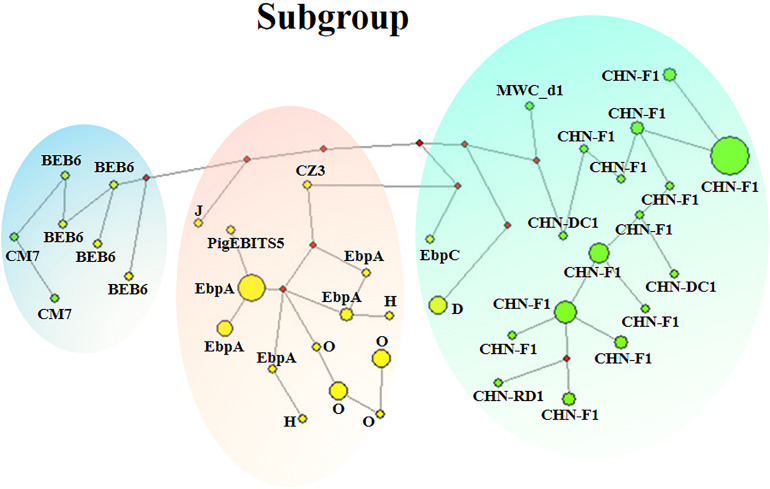
Median-joining network analysis for inferring intraspecific phylogenies of 90 *E. bieneusi* isolates belonging to 14 ITS genotypes from eight mammal species. The yellow and green colors in circles represent zoonotic ITS genotypes and host-adapted ITS genotypes, respectively. The diameter of each circle is proportional to the frequency of each multilocus genotype (MLG) on the basis of polymorphic/segregating sites of concatenated sequences of the five genetic loci including ITS. Length of lines connecting MLGs is proportional to the number of single nucleotide polymorphisms. ITS genotypes are labeled besides the circles. Three clusters marked in green, pink and blue are corresponded to subgroups 1–3, respectively.

Phylogenetic relationship of the *E. bieneus*i isolates was inferred by neighbor-joining analysis of the concatenated sequences of all the five genetic loci. Two phylogenetic clusters were observed, and the larger one was further divided into two subdivisions, leading to three subgroups altogether, reliability and confidence of which were verified based on the calculation of pairwise genetic distance (*F*_ST_) and gene flow (*Nm*) using DnaSP (Supplementary Table S5). Subgroup 1 was composed of MLGs from ITS genotypes CHN-DC1, CHN-F1, CHN-RD1, D, EbpC and MWC_d1 (in ITS group 1), mainly isolated from fur animals (blue foxes, arctic foxes, silver foxes and raccoon dogs). Subgroup 2 comprised MLGs from ITS genotypes CZ3, EbpA, H, O and PigEBITS5 (in ITS group 1) and J (in ITS group 2), which were mainly from pigs. Subgroup 3 consisted of MLGs from ITS genotype BEB6 and CM7 (in ITS group 2) derived from sheep. There was an absence of geographical segregation in *E. bieneusi* population in the present study (Figure 2). Meanwhile, the MLGs from zoonotic ITS genotypes were generally separated from the MLGs from host-adapted ones in ITS group 1 (Figure 2), which was also supported by median-joining network analysis of the entire concatenated multilocus sequences (Figure 3).

Subgroups 1–3 contained 23, 18, and seven MLGs derived from 52, 31 and seven *E. bieneusi* isolates, respectively. The *I*^S^_A_ values were positive for each of the three subgroups by LD analysis of the allelic profile data. Meanwhile, *V*_D_ values larger than the *L* values for subgroups 1–3 suggested the existence of LD and clonal population structures, with significant LD (*P*_MC_ < 0.01) for subgroups 1–3. Multilocus LD was recalculated when the same MLG treated as one individual. Subgroups 1, 2 both had positive *I*^S^_A_ values and *V*_D_ > *L* with significant *P*_MC_, confirming the existence of LD and clonal population structures. Due to no identical MLGs found in subgroup 3, the subgroup was not assessed again (Table 2).

## Discussion

MLST analysis has been widely used to study population genetics of *E. bieneusi* (Li and Xiao, 2019). In the present study, MLST analysis of the MS1, MS3, MS4, and MS7 loci was performed on 305 *E. bieneusi* isolates derived from various animal species. Differences in PCR amplification efficiencies were observed at the four different MLST loci: 50.16% at the MS1, 42.95% at the MS3, 43.61% at the MS4 and 41.97% at the MS7, which were all higher than those of *E. bieneusi* isolates in China from horses (6.67–18.67%) (Deng et al., 2016) and from Asiatic black bears (27.59–31.03%) (Deng et al., 2017), but lower than those of *E. bieneusi* isolates from NHPs (77.78–86.51%) (Karim et al., 2014) and from captive wild animals (76.74–86.05%) (Li et al., 2016a). Similar amplification efficiencies were found in one study of multilocus genotyping of *E. bieneusi* isolates from calves in China (38.36–61.64%) (Wang et al., 2016). The inconsistency of amplification efficiencies might be related to different ITS genotypes of *E. bieneusi* isolates in MLST analysis. In fact, micro-/mini-satellite sequences were searched in the whole-genome sequence database of the human-derived *E. bieneusi* isolate (H348), and the present PCR primers were designed for target genes (MS1, MS3, MS4, and MS7) based on nucleotide sequences flanking the potential micro-/mini-satellite repeats (Feng et al., 2011). Li et al. (2019b) has ever pointed out that hypermutation in *E. bieneusi* genome possibly hinders some isolates from effectively amplifying. Therefore, it is necessary to develop additional reliable and effective genetic markers that will successfully amplify isolates of all *E. bieneusi* genotypes to better understand their host specificity and population structure.

In the present study, 44 genotypes were obtained at the MS1 locus, followed by 26, 24, and 17 genotypes at the MS4, MS7, and MS3, respectively. Previous studies described different numerical ordinations of genotypes at the four loci (Feng et al., 2011; Li et al., 2013, 2017; Karim et al., 2014; Deng et al., 2016; Wan et al., 2016; Wang et al., 2018a, 2019; Wu et al., 2018). However, MS1 was generally observed to have the highest resolution in most studies, leading to more genotypes at this locus than the MS3, MS4, and MS7 loci. The appearance of this phenomenon might be related to the number and host origin of *E. bieneusi* isolates analyzed and the constitution of ITS genotypes.

In the present study, a total of 48 MLGs were identified out of 90 ITS-positive *E. bieneusi* isolates. The presence of significant LD and a low number of recombination events indicated a clonal population structure of *E. bieneusi* in the investigated areas. The results imply that MLGs of *E. bieneusi* are relatively stable in time and in space in the investigated areas, and MLST analysis can be used effectively in the longitudinal tracking of the transmission of *E. bieneusi* in the community and in investigation of outbreaks. To date, at least 11 studies have assessed the population structure of *E. bieneusi*, in which over 800 *E. bieneusi* isolates have been used for LD analysis, including over 100 human-derived isolates belonging to 14 ITS genotypes from Peru, India and Nigeria and over 700 animal-derived isolates belonging to 60 ITS genotypes from China and Kenya, and LD analysis revealed the wide existence of a clonal population structure (Li et al., 2012, 2013, 2016b, 2017, 2019; Karim et al., 2014; Wan et al., 2016; Wang et al., 2018a, 2019; Zhang et al., 2020; this study) (Table 4).

**TABLE 4:** *Enterocytozoon bieneusi* isolates used for LD analysis and characterizations of population structure and substructure.

Host (country)	*E. bieneusi* isolates used for LD analysis		No. of MLGs identified	Characterization		References
	Number	ITS genotypes		Population	Subgroup (n)	
Human (Peru)	72	A, D, EbpC, Peru7, Peru8, Peru10, Peru11, Type IV, WL11	39	Clonal	Clonal (*n* = 1); epidemic (*n* = 1)	Li et al., 2012
Human (Peru, India, Nigeria), Olive baboon (Kenya)	64^b^	A, D, EbpC, IH, Nig2, Nig3, Nig5, Peru7, Peru8, Peru10, Peru11, PigEBITS7, Type IV, WL11	48	Clonal^d^	Clonal (*n* = 1); epidemic (*n* = 1)	Li et al., 2013
NHP (China)	85	CM2, D, Henan-V, Macaque3, Peru8, Peru11, PigEBITS7, Type IV	59	Clonal	Epidemic (*n* = 4)	Karim et al., 2014
Fox, raccoon dog^a^ (China)	39	D	10	Clonal	NA	Li et al., 2016b
Pig (China)	101	CHN7, CS-4, EbpA, EbpB, EbpC, LW1, Henan-IV, O, PigEBITS3	44	Clonal	Clonal (*n* = 1); epidemic (*n* = 2)	Wan et al., 2016
	109^c^	CHC5, CHG3, CHN7, CS-4, D, EbpA, H, Henan-IV, PigEb4, PigEBITS4, PigEBITS5, SHZA1, SHZC1, SLTC1-SLTC3, SMXB1, SMXC1, SMXD1, SMXD2, SYLA1-SYLA5, SYLC1, SYLD1, SZZA1, SZZA2, SZZB1, SZZC1, SZZD1, SZZD2	87	Clonal	NA	Wang et al., 2018a
	93	EbpC, EbpA, H, PigEBITS4	76	Clonal	NA	Li et al., 2019
	52	CM11, D, EbpC, EbpA, FJF	48	Clonal	NA	Zhang et al., 2020
Dairy cattle (China)	106	BEB4, I, J	71	Clonal	NA	Wang et al., 2019
Giant panda (China)	36	D, SC02, SC05, SC06	24	Clonal	Clonal (*n* = 1); epidemic (*n* = 2)	Li et al., 2017
Dairy cattle, pig, sheep, sika deer, blue fox, arctic fox, silver fox, raccoon dog (China)	90	BEB6, CHN-DC1, CHN-F1, CHN-RD1, CM7, CZ3, D, EbpA, EbpC, H, J, MWC_d1, O, PigEBITS5	48	Clonal	Clonal (*n* = 3); epidemic (*n* = 1)	This study

*Five genotypes in Table 4 have been changed to the first published names instead of genotype names described in original papers: CM1 to Macaque3 (Karim et al., 2014); Henan-I to LW1 (Wan et al., 2016); CHG19 to PigEBITS4 (Wang et al., 2018a; Li et al., 2019); CHS5 to EbpA (Wang et al., 2018a; Zhang et al., 2020); CM6 to CS-4 (Wang et al., 2018a). ^a^39 E. bieneusi isolates are derived from foxes (n = 30) and raccoon dogs (n = 9). ^b^64 E. bieneusi isolates used for LD analysis are derived from humans (n = 59) in Peru, India, Nigeria and olive baboons (n = 5) in Kenya. Among them, MLST data of 26 human-derived isolates are from a study conducted in Peru by Li et al. (2012). ^c^These ITS genotypes are used for MLST analysis without specific information of genotypes used for LD analysis. ^d^A clonal population structure is observed in each of 3 geographical locations (Peru, India, and Nigeria).*

Based on the present result of phylogenetic analysis of MLGs, genetic segregation related to hosts was observed at a large extent in the *E. bieneusi* population from various mammal species, with subgroups 1–3 composed of MLGs mainly from fur animals, pigs and sheep, respectively. In fact, host adaptation has been reported in some studies of *E. bieneusi* populations: fur animals (foxes and raccoon dogs) versus humans (Li et al., 2016b); pigs versus others (humans, NHPs, cattle, horses, and bears) (Li et al., 2019); dairy cattle versus others (humans, NHPs, pigs, bears, horses and kangaroos) (Wang et al., 2019). These results will be helpful to understand zoonotic or interspecies transmission of *E. bieneusi.*

In the present study, the *E. bieneusi* MLGs were not observed to be related to geographical locations of DNA specimens. This result was similar to some previous studies of *E. bieneusi* populations from humans in Peru, India and Nigeria (Li et al., 2013) and from animals in China (Li et al., 2017; Wang et al., 2019; Zhang et al., 2020). However, genetic segregation at some geographical level appeared in the other studies conducted in China (Wan et al., 2016; Wang et al., 2018a; Li et al., 2019). No geographical segregation of *E. bieneusi* isolates was seen in the investigated areas, which might be related to smaller geographical areas and number of *E. bieneusi* isolates as well as fewer ITS genotypes analyzed.

To date, approximately 500 *E. bieneusi* ITS genotypes have been identified: ∼60 only found in humans; ∼390 only found in animals; ∼50 found in both humans and animals (Li et al., 2019b; Shen et al., 2020). However, the genetic reasons behind this observation of *E. bieneusi* genotypes remain unclear, especially for those genotypes belonging to group 1 with zoonotic nature and group 2 with increasing importance for public health. In the present study, 48 MLGs belonging to 14 ITS genotypes were divided into three different subgroups. The MLGs were generally separated by zoonotic potential, revealing possible genetic segregation of *E. bieneusi* population. Subgroup 1 was composed of the MLGs from zoonotic ITS genotypes D and EbpC with wide host ranges and those from host-adapted ITS genotypes (CHN-DC1, CHN-F1, CHN-RD1 and MWC_d1) with narrow host ranges based on the summarized data in Supplementary Table S6. Meanwhile, the MLG from ITS genotype J fell into subgroup 2 consisting of those from zoonotic ITS genotypes CZ3, EbpA, H, O and PigEBITS5. Combined with network analysis, subgroups 1, 3 might originate from subgroup 2, with D and EbpC, and J as evolutionary transitions, respectively. It was speculated that there might be different *E. bieneusi* populations with different genetic structures and public health potential in their evoluting processes, with the possible direction from zoonotic genotypes to host-adapted genotypes. Finally, some genotypes of *E. bieneusi* might be adapted to changing host environments, and were successfully chosen by hosts. Nevertheless, it is still unclear on the possible factors promoting adaptation of different *E. bieneusi* genotypes to specific hosts or host changes.

## Conclusion

The present study describes the genetic characterizations of *E. bieneusi* isolates derived from various mammal and bird species at the four MLST loci (MS1, MS3, MS4, and MS7) in northeastern China. 48 MLGs were produced out of 14 ITS genotypes, revealing MLGs have a higher genetic diversity than ITS genotypes. MLST data and a clonal population structure of *E. bieneusi* will aid in the longitudinal tracking of the attribution of source of infection/contamination and in elucidating transmission dynamics, and will provide valuable information for making efficient control strategies to intervene with and prevent occurrence of microsporidiosis caused by *E. bieneusi* among animals and transmission of *E. bieneusi* from animals to humans in the investigated areas. The result of host adaptation of *E. bieneusi* population has a reference effect on further understanding of zoonotic or interspecies transmission of *E. bieneusi.* The observation of genetic segregation of *E. bieneusi* population by zoonotic potential and possible evolution direction of *E. bieneusi* from zoonotic genotypes to host-adapted genotypes will improve our understanding of evolution of *E. bieneusi* population. However, the role of host, geographical, and temporal factors in the evolution mechanism of *E. bieneusi* populations needs to be characterized in the future.

## Data Availability Statement

The datasets presented in this study can be found in online repositories. The names of the repository/repositories and accession number(s) can be found in the article/Supplementary Material.

## Ethics Statement

Ethical review and approval was not required for the animal study because all the specimens analyzed were DNA preparations from animal feces.

## Author Contributions

AL and YS designed this study and made the final revision. XL and YW performed the experiments, wrote the first draft of the manuscript, and prepared the tables and figures. XL, YW, BG, FY, YJ, and KZ analyzed the data. WZ and JC contributed to the reagents and materials. All authors contributed to the article and approved the submitted version.

## Conflict of Interest

The authors declare that the research was conducted in the absence of any commercial or financial relationships that could be construed as a potential conflict of interest.

## Footnotes

1 http://www.ncbi.nlm.nih.gov/blast/

2 http://www.clustal.org/

3 http://guanine.evolbio.mpg.de/cgi-bin/lian/lian.cgi.pl

4 http://www.ub.es/dnasp/

5 http://www.megasoftware.net/

6 http://www.fluxus-engineering.com/sharenet_rn.htm
