# Supplementary material for: Multilocus Sequence Typing of Enterocytozoon bieneusi Isolates From Various Mammal and Bird Species and Assessment of Population Structure and Substructure
Source: Front Microbiol. 2020 Jun 26;11:1406. doi: 10.3389/fmicb.2020.01406 (PMC7333453; doi:10.3389/fmicb.2020.01406)
Supplement: Supplementary file 1 [file Data_Sheet_1.doc]

**Supplementary Table S1 Studies on ITS genotypes of *E. bieneusi* used for multilocus sequence typing analysis.**

| **Host** | **Country** | **Genotype^a^** | **Reference** |
| --- | --- | --- | --- |
| **human** | | | |
| HIV-positive adult; child | Brazil, Peru | A, D, NIA1, Peru6, Peru10, Peru11, Peru15, Type IV, WL12 | Feng et al., 2011 |
| HIV-positive patient | Peru | A, D, EbpC, Peru7, Peru8, Peru10, Peru11, Type IV, WL11 | Li et al., 2012 |
| HIV-positive patient | Peru, India, Nigeria | A, D, EbpC, IH, Nig2, Nig3, Nig5, Peru7, Peru8, Peru10, Peru11, PigEBITS7, Type IV, WL11 | Li et al., 2013 |
| diarrheic child | Uganda | Unspecific | Widmer et al., 2013 |
| HIV-positive person; solidorgan transplant recipient; patient with hematologic malignancies, autoimmune diseases or cirrhosis | France | C, C-like01, C-like04, SC9, Type IV, Type IV-like01 | Desoubeaux et al., 2019 |
| **animal** | | | |
| alpaca | China | ALP1, ALP3, P, SH11 | Ma et al., 2019 |
| Asiatic black bear | China | ABB2, Horse2, CHB1, SC02 | Deng et al., 2017 |
| Asiatic black bear | China | CHB1, D, MJ1-MJ13, SC01, SC02 | Wu J et al., 2018 |
| dairy and native beef calf | China | BEB4, CSX1, CSX2, I, J | Wang et al., 2016 |
| pre-weaned Holstein calf | China | BEB4, J | Tang et al., 2018 |
| dairy cattle | China | BEB4, BEB8, BEB10, CM21, CGC1-CGC3, D, EbpC, I, J | Wang et al., 2019 |
| forest musk deer | China | EbpC | Song et al., 2018 |
| fox | China | CHN-DC1, D, NCF5, Peru8, Type IV, WildBoar3 | Zhang et al., 2016 |
| fox; raccoon dog | China | D | Li et al., 2016a |
| giant panda; red panda | China | EbpC, I-like | Tian et al., 2015 |
| giant panda | China | CHB1, D, EbpA, EbpC, Peru6, SC01, SC02, SC04-SC08 | Li et al., 2017 |
| horse | China | D, Horse1, Horse2, SC02, SCH1-SCH4, YNH1, YNH2 | Deng et al., 2016a |
| NHP | China | CM2, CM3, D, Henan-V, Macaque3, Peru8, Peru11, PigEBITS7, Type IV | Karim et al., 2014 |
| NHP | China | D, Horse1, PigEBITS7, SC02 | Zhong et al., 2017a |
| olive baboon | Kenya | D | Li et al., 2013 |
| pig; chicken; marmoset; guinea pig; muskrat; raccoon; cat; dog; cattle; goat | Peru, Portugal, USA | BEB6, EbpC, J, Peru8, Peru16, PtEb VIII, PtEb IX, PtEb XI, PtEb XII, WL1-WL4 | Feng et al., 2011 |
| pig | China | CHN7, CS-4, EbpA, EbpB, EbpC, LW1, Henan-IV, O, PigEBITS3 | Wan et al., 2016 |
| pig | China | CHC5, CHG3, CHN7, CS-4, D, EbpA, H, Henan-IV, PigEb4, PigEBITS4, PigEBITS5, SHZA1, SHZC1, SLTC1-SLTC3, SMXB1, SMXC1, SMXD1, SMXD2, SYLA1-SYLA5, SYLC1, SYLD1, SZZA1, SZZA2, SZZB1, SZZC1, SZZD1, SZZD2 | Wang et al., 2018 |
| pig | China | CAF1, D, EbpC, EbpA, G, GD1, H, Henan-IV, PigEBITS5, YN1-YN3, ZJ1, ZJ2 | Zou et al., 2018 |
| pig | China | CHC5, D, EbpC, EbpA, H, Henan-III, I, PigEBITS4, XZP-I, XZP-II | Li et al., 2019 |
| pig | China | CAF1, CHG7, CHN-RR2, CM11, D, EbpA, EbpC, FJF, FJS, G, PigEBITS5 | Zhang et al., 2020 |
| Tibetan pig | China | EbpC, Henan-IV, SCT01, SCT02 | Luo et al., 2019 |
| Tibetan pig | China | CS-8, EbpC, EbpD, GB11, GB31, PigEBITS5 | Zou et al., 2019 |
| red-bellied tree squirrel | China | CE01, D, Horse2, EbpC, SC02 | Deng et al., 2016b |
| red kangaroo | China | CHK1, CSK1, CSK2 | Zhong et al., 2017b |
| black-boned sheep; black-boned goat | China | BEB6, CHG1, CHG3, CM7, CM21, COS-II, PigEb4, EbpA, EbpC, SX1, YNS1, YSM1 | Chen et al., 2018 |
| Tibetan sheep | China | BEB6, CGS1, CHS3, CM7 | Wu Y et al., 2018 |
| golden takin | China | BEB6, D, I, TEB1-TEB4 | Zhao et al., 2015 |
| wildlife | China | BEB6, CHB1, CHS9, D, EbpC, Peru6, SC01, SC02 | Li et al., 2016b |
| wild rat | China | D, Peru8, PigEBITS7, Type IV, CQR-1, CQR-2, CQR-3, GDR-1, GDR-2, GDR-3 | Gui et al., 2020 |

*24 genotypes in* ***Supplementary Table S1*** *have been changed to the first published names instead of genotype names described in original papers: ABB1 to CHB1 (Deng et al., 2017); CE02 to Horse2 (Deng et al., 2016b); CHG19 to PigEBITS4 (Wang et al., 2018; Li et al., 2019); CHG23 to EbpC (Zhang et al., 2020); CHN1 to BEB4 (Wang et al., 2016); CHS12 to CS-8 (Zou et al., 2019); CHS5 to EbpA (Chen et al., 2018; Wang et al., 2018; Zou et al., 2018; Zhang et al., 2020); CM1 to Macaque3 (Karim et al., 2014); CM19 to BEB8 (Wang et al., 2019); CM6 to CS-4 (Wang et al., 2018); COS-I to CM7 (Chen et al., 2018); F to EbpA (Li et al., 2017); Henan-I to LW1 (Wan et al., 2016); K to Type IV (Gui et al., 2020); KIN-1 to CAF1 (Zou et al., 2018; Zhang et al., 2020); SC03 to EbpC (Li et al., 2016b; Song et al., 2018); SCM01 to Horse1 (Zhong et al., 2017a); WL5 to WL4 (Feng et al., 2011); NCF1-NCF4 to WildBoar3 (Zhang et al., 2016); NCF6 to NCF5 (Zhang et al., 2016); NCF7 to D (Zhang et al., 2016).*

**Supplementary Table S2 Distribution of ITS genotypes (n = 28) of *E. bieneusi* isolates (n = 305) analyzed in this study by host and location and PCR amplification efficiency at the four MLST loci.**

| **Phylogenetic group** | **ITS genotype (n)** | **Host (n)** | **Location (n)** | **Amplification efficiency (%)** | | | | **No. of specimens^a^** |
| --- | --- | --- | --- | --- | --- | --- | --- | --- |
|  |  |  |  | **MS1** | **MS3** | **MS4** | **MS7** |  |
| 1 | CAF1 (1) | sika deer (1) | Suihua (1) | 0/1 (0) | 0/1 (0) | 0/1 (0) | 0/1 (0) | 0 |
|  | CHN-DC1 (4) | blue fox (2) | Harbin (1) | 0/1 (0) | 1/1 (100) | 1/1 (100) | 0/1 (0) | 0 |
|  |  |  | Suihua (1) | 0/1 (0) | 0/1 (0) | 0/1 (0) | 0/1 (0) | 0 |
|  |  | dairy cattle (1) | Harbin (1) | 1/1 (100) | 1/1 (100) | 1/1 (100) | 1/1 (100) | 1 |
|  |  | silver fox (1) | Suihua (1) | 1/1 (100) | 1/1 (100) | 1/1 (100) | 1/1 (100) | 1 |
|  | CHN-F1 (79) | arctic fox (16) | Suihua (16) | 14/16 (87.50) | 13/16 (81.25) | 12/16 (75.00) | 12/16 (75.00) | 11 |
|  |  | blue fox (59) | Harbin (25) | 12/25 (48.00) | 11/25 (44.00) | 10/25 (40.00) | 11/25 (44.00) | 8 |
|  |  |  | Suihua (34) | 28/34 (82.35) | 29/34 (85.29) | 27/34 (79.41) | 26/34 (76.47) | 23 |
|  |  | raccoon dog (2) | Harbin (2) | 1/2 (50.00) | 1/2 (50.00) | 1/2 (50.00) | 1/2 (50.00) | 1 |
|  |  | silver fox (2) | Suihua (2) | 2/2 (100) | 0/2 (0) | 2/2 (100) | 0/2 (0) | 0 |
|  | CHN-RD1 (14) | reindeer (13) | Great Hinggan Mountains (13) | 6/13 (46.15) | 6/13 (46.15) | 0/13 (0) | 6/13 (46.15) | 0 |
|  |  | sika deer (1) | Suihua (1) | 1/1 (100) | 1/1 (100) | 1/1 (100) | 1/1 (100) | 1 |
|  | CZ3 (1) | pig (1) | Harbin (1) | 1/1 (100) | 1/1 (100) | 1/1 (100) | 1/1 (100) | 1 |
|  | D (12) | blue fox (2) | Harbin (2) | 0/2 (0) | 0/2 (0) | 0/2 (0) | 0/2 (0) | 0 |
|  |  | brown rat (2) | Harbin (2) | 1/2 (50.00) | 0/2 (0) | 0/2 (0) | 0/2 (0) | 0 |
|  |  | Common crane (1) | Qiqihar (1) | 0/1 (0) | 0/1 (0) | 0/1 (0) | 0/1 (0) | 0 |
|  |  | dairy cattle (1) | Harbin (1) | 1/1 (100) | 1/1 (100) | 1/1 (100) | 1/1 (100) | 1 |
|  |  | pig (4) | Suihua (4) | 3/4 (75.00) | 3/4 (75.00) | 2/4 (50.00) | 3/4 (75.00) | 2 |
|  |  | raccoon dog (1) | Harbin (1) | 0/1 (0) | 0/1 (0) | 0/1 (0) | 0/1 (0) | 0 |
|  |  | sheep (1) | Suihua (1) | 1/1 (100) | 1/1 (100) | 1/1 (100) | 1/1 (100) | 1 |
|  | EbpA (24) | pig (23) | Harbin (23) | 18/23 (78.26) | 19/23 (82.61) | 16/23 (69.57) | 18/23 (78.26) | 16 |
|  |  | reindeer (1) | Great Hinggan Mountains (1) | 0/1 (0) | 0/1 (0) | 0/1 (0) | 0/1 (0) | 0 |
|  | EbpC (2) | brown rat (1) | Harbin (1) | 0/1 (0) | 0/1 (0) | 0/1 (0) | 0/1 (0) | 0 |
|  |  | pig (1) | Harbin (1) | 1/1 (100) | 1/1 (100) | 1/1 (100) | 1/1 (100) | 1 |
|  | ESH-02 (1) | chicken (1) | Suihua (1) | 1/1 (100) | 1/1 (100) | 0/1 (0) | 0/1 (0) | 0 |
|  | H (4) | pig (4) | Harbin (4) | 4/4 (100) | 3/4 (75.00) | 2/4 (50) | 3/4 (75) | 2 |
|  | HLJ-CP1 (1) | brown rat (1) | Harbin (1) | 1/1 (100) | 1/1 (100) | 1/1 (100) | 0/1 (0) | 0 |
|  | JLD-IV (1) | sika deer (1) | Suihua (1) | 0/1 (0) | 1/1 (100) | 1/1 (100) | 0/1 (0) | 0 |
|  | LW1 (1) | pig (1) | Harbin (1) | 0/1 (0) | 0/1 (0) | 0/1 (0) | 1/1 (100) | 0 |
|  | MWC_d1 (3) | red deer (2) | Great Hinggan Mountains (2) | 1/2 (50.00) | 0/2 (0) | 1/2 (50.00) | 0/2 (0) | 0 |
|  |  | sika deer (1) | Suihua (1) | 1/1 (100) | 1/1 (100) | 1/1 (100) | 1/1 (100) | 1 |
|  | NCR1 (3) | arctic fox (3) | Suihua (3) | 1/3 (33.33) | 0/3 (0) | 0/3 (0) | 0/3 (0) | 0 |
|  | O (17) | pig (2) | Harbin (2) | 1/2 (50.00) | 2/2 (100) | 1/2 (50.00) | 2/2 (100) | 1 |
|  |  | pig (15) | Suihua (15) | 11/15 (73.33) | 13/15 (86.67) | 10/15 (66.67) | 10/15 (66.67 ) | 9 |
|  | Peru6 (58) | chicken (33) | Suihua (33) | 2/33 (6.06) | 0/33 (0) | 0/33 (0) | 1/33 (3.03) | 0 |
|  |  | duck (1) | Suihua (1) | 0/1 (0) | 0/1 (0) | 0/1 (0) | 0/1 (0) | 0 |
|  |  | goose (7) | Suihua (7) | 0/7 (0) | 0/7 (0) | 0/7 (0) | 0/7 (0) | 0 |
|  |  | horse (1) | Daqing (1) | 0/1 (0) | 0/1 (0) | 0/1 (0) | 0/1 (0) | 0 |
|  |  | pigeon (7) | Suihua (7) | 4/7 (57.14) | 0/7 (0) | 5/7 (71.43) | 5/7 (71.43) | 0 |
|  |  | rex rabbit (4) | Harbin (4) | 0/4 (0) | 0/4 (0) | 0/4 (0) | 0/4 (0) | 0 |
|  |  | reindeer (2) | Great Hinggan Mountains (2) | 0/2 (0) | 0/2 (0) | 0/2 (0) | 0/2 (0) | 0 |
|  |  | sheep (3) | Suihua (3) | 1/3 (33.33) | 0/3 (0) | 0/3 (0) | 0/3 (0) | 0 |
|  | PigEBITS5 (1) | pig (1) | Harbin (1) | 1/1 (100) | 1/1 (100) | 1/1 (100) | 1/1 (100) | 1 |
|  | Type IV (6) | dairy cattle (1) | Harbin (1) | 0/1 (0) | 0/1 (0) | 0/1 (0) | 0/1 (0) | 0 |
|  |  | horse (1) | Daqing (1) | 0/1 (0) | 0/1 (0) | 0/1 (0) | 0/1 (0) | 0 |
|  |  | reindeer (4) | Great Hinggan Mountains (4) | 0/4 (0) | 0/4 (0) | 0/4 (0) | 0/4 (0) | 0 |
|  | WildBoar3 (8) | arctic fox (2) | Suihua (2) | 0/2 (0) | 1/2 (50.00) | 1/2 (50.00) | 0/2 (0) | 0 |
|  |  | blue fox (2) | Suihua (2) | 0/2 (0) | 0/2 (0) | 0/2 (0) | 0/2 (0) | 0 |
|  |  | silver fox (4) | Suihua (4) | 0/4 (0) | 0/4 (0) | 2/4 (50.00) | 0/4 (0) | 0 |
| 2 | BEB6 (36) | goose (3) | Suihua (3) | 2/3 (66.67) | 0/3 (0) | 3/3 (100) | 0/3 (0) | 0 |
|  |  | red deer (5) | Great Hinggan Mountains (5) | 1/5 (20.00) | 1/5 (20.00) | 1/5 (20.00) | 2/5 (40.00) | 0 |
|  |  | sheep (25) | Suihua (18) | 10/18 (55.56) | 4/18 (22.22) | 7/18 (38.89) | 7/18 (38.89) | 3 |
|  |  |  | Qiqihar (7) | 3/7 (42.86) | 2/7 (28.57) | 2/7 (28.57) | 3/7 (42.86) | 2 |
|  |  | sika deer (3) | Suihua (3) | 0/3 (0) | 0/3 (0) | 0/3 (0) | 0/3 (0) | 0 |
|  | CHG1 (1) | sheep (1) | Suihua (1) | 0/1 (0) | 0/1 (0) | 0/1 (0) | 0/1 (0) | 0 |
|  | CM7 (4) | sheep (4) | Suihua (4) | 4/4 (100) | 2/4 (50.00) | 4/4 (100) | 3/4 (75.00) | 2 |
|  | COS-II (2) | sheep (2) | Suihua (2) | 1/2 (50.00) | 0/2 (0) | 0/2 (0) | 1/2 (50.00) | 0 |
|  | HLJD-I (2) | sika deer (2) | Suihua (2) | 2/2 (100) | 0/2 (0) | 1/2 (50.00) | 1/2 (50.00) | 0 |
|  | I (11) | dairy cattle (9) | Harbin (9) | 6/9 (66.67) | 5/9 (55.56) | 7/9 (77.78) | 1/9 (11.11) | 0 |
|  |  | rex rabbit (2) | Harbin (2) | 0/2 (0) | 0/2 (0) | 2/2 (100) | 0/2 (100) | 0 |
|  | J (6) | dairy cattle (5) | Harbin (5) | 2/5 (40) | 3/5 (60) | 2/5 (40) | 1/5 (20) | 1 |
|  |  | red deer (1) | Great Hinggan Mountains (1) | 0/1 (0) | 0/1 (0) | 0/1 (0) | 0/1 (0) | 0 |
| 9 | WR8 (2) | brown rat (2) | Harbin (2) | 1/2 (50.00) | 0/2 (0) | 0/2 (0) | 1/2 (50.00) | 0 |

*^a^The specimens were successfully amplified and sequenced at the four Loci (MS1,MS3, MS7, MS7) and would be used for LD analysis in this study.*

**Supplementary Table S3 Host distributions and phylogenetic groups of ITS genotypes of *E. bieneusi.***

| **Host (n)** | **ITS genotypes** | |
| --- | --- | --- |
|  | **Group 1 (n)** | **Other groups (n)^a^** |
| **mammal (252)** | | |
| pig (52) | CZ3 (1), D (4), EbpA (23), EbpC (1), H (4), LW1 (1), O (17), PigEBITS5 (1) |  |
| dairy cattle (17) | CHN-DC1 (1), D (1), Type IV (1) | I (9), J (5) |
| sheep (36) | D (1), Peru6 (3) | BEB6 (25), CHG1 (1), CM7 (4), COS-II (2) |
| sika deer (9) | CAF1 (1), CHN-RD1 (1), JLD-IV (1), MWC_d1 (1) | BEB6 (3), HLJD-I (2) |
| red deer (8) | MWC_d1 (2) | BEB6 (5), J (1) |
| reindeer (20) | CHN-RD1 (13), EbpA (1), Peru6 (2), Type IV (4) |  |
| blue fox (65) | CHN-DC1 (2), CHN-F1 (59), D (2), WildBoar3 (2) |  |
| arctic fox (20) | CHN-F1 (16), NCR1 (2), WildBoar3 (2) |  |
| silver fox (8) | CHN-DC1 (1), CHN-F1 (2), NCR1 (1), WildBoar3 (4) |  |
| raccoon dog (3) | D (1), CHN-F1 (2) |  |
| rex rabbit (6) | Peru6 (4) | I (2) |
| horse (2) | Peru6 (1), Type IV (1) |  |
| brown rat (6) | HLJ-CP1 (1), EbpC (1), D (2) | WR8 (2) |
| **bird (53)** |  |  |
| chicken (34) | Peru6 (33), ESH-02 (1) |  |
| duck (1) | Peru6 (1) |  |
| goose (10) | Peru6 (7) | BEB6 (3) |
| pigeon (7) | Peru6 (7) |  |
| Common crane (1) | D (1) |  |

***^a^****All the genotypes in this column are in group 2 except genotype WR8 in group 9.*

**Supplementary Table S4 Multilocus genotypes of *E. bieneusi* isolates used for LD analysis in the present study**.

| **Phylogenetic group** | **ITS genotype (n)** | **Host (n)** | **Location (n)** | **Code** | **GenBank accession number** | | | | | **MLG** |
| --- | --- | --- | --- | --- | --- | --- | --- | --- | --- | --- |
|  |  |  |  |  | **MS1** | **MS3** | **MS4** | **MS7** | **ITS** |  |
| 1 | CHN-DC1 (2) | silver fox (1) | Suihua | 205 | MT267442 | MT267470 | MT267383 | MT267394 | KJ710333 | MLG1 |
|  |  | dairy cattle (1) | Harbin | 295 | MT267442 | MT267470 | MT267382 | MT267407 | KJ710333 | MLG2 |
|  | CHN-F1 (43) | blue fox (31) | Harbin | 136 | MT267442 | MT267470 | MT267378 | MT267405 | KR998501 | MLG3 |
|  |  |  | Harbin | 139 | MT267442 | MT267470 | MT267378 | MT267406 | KR998501 | MLG4 |
|  |  |  | Harbin | 142 | MT267442 | MT267470 | MT267378 | MT267405 | KR998501 | MLG3 |
|  |  |  | Harbin | 147 | MT267442 | MT267470 | MT267366 | MT267406 | KR998501 | MLG5 |
|  |  |  | Harbin | 148 | MT267442 | MT267470 | MT267379 | MT267406 | KR998501 | MLG6 |
|  |  |  | Harbin | 149 | MT267442 | MT267470 | MT267380 | MT267406 | KR998501 | MLG7 |
|  |  |  | Harbin | 151 | MT267442 | MT267470 | MT267380 | MT267406 | KR998501 | MLG7 |
|  |  |  | Harbin | 152 | MT267442 | MT267470 | MT267380 | MT267406 | KR998501 | MLG7 |
|  |  |  | Suihua | 153 | MT267442 | MT267470 | MT267380 | MT267406 | KR998501 | MLG7 |
|  |  |  | Suihua | 155 | MT267442 | MT267470 | MT267380 | MT267406 | KR998501 | MLG7 |
|  |  |  | Suihua | 158 | MT267442 | MT267470 | MT267380 | MT267407 | KR998501 | MLG8 |
|  |  |  | Suihua | 159 | MT267442 | MT267470 | MT267381 | MT267406 | KR998501 | MLG9 |
|  |  |  | Suihua | 161 | MT267442 | MT267470 | MT267380 | MT267406 | KR998501 | MLG7 |
|  |  |  | Suihua | 162 | MT267442 | MT267470 | MT267380 | MT267406 | KR998501 | MLG7 |
|  |  |  | Suihua | 163 | MT267442 | MT267470 | MT267380 | MT267406 | KR998501 | MLG7 |
|  |  |  | Suihua | 164 | MT267442 | MT267470 | MT267380 | MT267406 | KR998501 | MLG7 |
|  |  |  | Suihua | 166 | MT267442 | MT267470 | MT267380 | MT267406 | KR998501 | MLG7 |
|  |  |  | Suihua | 168 | MT267442 | MT267470 | MT267380 | MT267406 | KR998501 | MLG7 |
|  |  |  | Suihua | 170 | MT267442 | MT267470 | MT267380 | MT267406 | KR998501 | MLG7 |
|  |  |  | Suihua | 171 | MT267442 | MT267470 | MT267380 | MT267406 | KR998501 | MLG7 |
|  |  |  | Suihua | 174 | MT267442 | MT267470 | MT267380 | MT267406 | KR998501 | MLG7 |
|  |  |  | Suihua | 175 | MT267442 | MT267470 | MT267379 | MT267406 | KR998501 | MLG6 |
|  |  |  | Suihua | 176 | MT267442 | MT267470 | MT267380 | MT267406 | KR998501 | MLG7 |
|  |  |  | Suihua | 177 | MT267442 | MT267470 | MT267380 | MT267406 | KR998501 | MLG7 |
|  |  |  | Suihua | 178 | MT267442 | MT267470 | MT267380 | MT267407 | KR998501 | MLG8 |
|  |  |  | Suihua | 179 | MT267442 | MT267470 | MT267380 | MT267406 | KR998501 | MLG7 |
|  |  |  | Suihua | 180 | MT267442 | MT267470 | MT267380 | MT267406 | KR998501 | MLG7 |
|  |  |  | Suihua | 212 | MT267442 | MT267470 | MT267382 | MT267405 | KR998501 | MLG10 |
|  |  |  | Suihua | 213 | MT267442 | MT267473 | MT267382 | MT267408 | KR998501 | MLG11 |
|  |  |  | Suihua | 218 | MT267442 | MT267470 | MT267382 | MT267405 | KR998501 | MLG10 |
|  |  |  | Suihua | 219 | MT267442 | MT267470 | MT267382 | MT267407 | KR998501 | MLG12 |
|  |  | arctic fox (11) | Suihua | 182 | MT267444 | MT267470 | MT267382 | MT267405 | KR998501 | MLG13 |
|  |  |  | Suihua | 185 | MT267444 | MT267470 | MT267383 | MT267408 | KR998501 | MLG14 |
|  |  |  | Suihua | 187 | MT267442 | MT267470 | MT267382 | MT267405 | KR998501 | MLG10 |
|  |  |  | Suihua | 188 | MT267442 | MT267470 | MT267383 | MT267408 | KR998501 | MLG15 |
|  |  |  | Suihua | 189 | MT267442 | MT267470 | MT267384 | MT267405 | KR998501 | MLG16 |
|  |  |  | Suihua | 190 | MT267443 | MT267470 | MT267384 | MT267405 | KR998501 | MLG17 |
|  |  |  | Suihua | 192 | MT267443 | MT267470 | MT267384 | MT267405 | KR998501 | MLG17 |
|  |  |  | Suihua | 193 | MT267442 | MT267470 | MT267384 | MT267405 | KR998501 | MLG16 |
|  |  |  | Suihua | 194 | MT267443 | MT267470 | MT267384 | MT267405 | KR998501 | MLG17 |
|  |  |  | Suihua | 195 | MT267442 | MT267472 | MT267384 | MT267406 | KR998501 | MLG18 |
|  |  |  | Suihua | 196 | MT267442 | MT267470 | MT267384 | MT267405 | KR998501 | MLG16 |
|  |  | raccoon dog (1) | Harbin | 203 | MT267442 | MT267470 | MT267382 | MT267405 | KR998501 | MLG10 |
|  | CHN-RD1 (1) | sika deer (1) | Suihua | 097 | MT267438 | MT267469 | MT267375 | MT267400 | KR632538 | MLG19 |
|  | CZ3 (1) | pig (1) | Harbin | 066 | MT267422 | MT267465 | MT267372 | MT267395 | GU198951 | MLG20 |
|  | D (4) | pig (2) | Suihua | 115 | MT267419 | MT267460 | MT267365 | MT267394 | AF101200 | MLG21 |
|  |  |  | Suihua | 281 | MT267419 | MT267460 | MT267365 | MT267394 | AF101200 | MLG21 |
|  |  | sheep (1) | Suihua | 030 | MT267419 | MT267460 | MT267365 | MT267394 | AF101200 | MLG21 |
|  |  | dairy cattle (1) | Harbin | 287 | MT267427 | MT267460 | MT267366 | MT267407 | AF101200 | MLG22 |
|  | EbpA (16) | pig (16) | Harbin | 038 | MT267423 | MT267465 | MT267367 | MT267396 | AF076040 | MLG23 |
|  |  |  | Harbin | 039 | MT267423 | MT267465 | MT267367 | MT267396 | AF076040 | MLG23 |
|  |  |  | Harbin | 041 | MT267423 | MT267465 | MT267367 | MT267396 | AF076040 | MLG23 |
|  |  |  | Harbin | 043 | MT267423 | MT267465 | MT267367 | MT267396 | AF076040 | MLG23 |
|  |  |  | Harbin | 046 | MT267423 | MT267465 | MT267367 | MT267396 | AF076040 | MLG23 |
|  |  |  | Harbin | 048 | MT267425 | MT267465 | MT267369 | MT267395 | AF076040 | MLG24 |
|  |  |  | Harbin | 049 | MT267423 | MT267465 | MT267367 | MT267396 | AF076040 | MLG23 |
|  |  |  | Harbin | 050 | MT267426 | MT267465 | MT267369 | MT267399 | AF076040 | MLG25 |
|  |  |  | Harbin | 051 | MT267423 | MT267465 | MT267367 | MT267398 | AF076040 | MLG26 |
|  |  |  | Harbin | 060 | MT267428 | MT267465 | MT267370 | MT267399 | AF076040 | MLG27 |
|  |  |  | Harbin | 063 | MT267431 | MT267465 | MT267369 | MT267399 | AF076040 | MLG28 |
|  |  |  | Harbin | 065 | MT267423 | MT267465 | MT267367 | MT267396 | AF076040 | MLG23 |
|  |  |  | Harbin | 067 | MT267432 | MT267465 | MT267367 | MT267399 | AF076040 | MLG29 |
|  |  |  | Harbin | 068 | MT267423 | MT267465 | MT267367 | MT267396 | AF076040 | MLG23 |
|  |  |  | Harbin | 077 | MT267423 | MT267465 | MT267367 | MT267398 | AF076040 | MLG26 |
|  |  |  | Harbin | 083 | MT267423 | MT267465 | MT267367 | MT267397 | AF076040 | MLG30 |
|  | EbpC (1) | pig (1) | Harbin | 036 | MT267422 | MT267464 | MT267366 | MT267395 | AF076042 | MLG31 |
|  | H (2) | pig (2) | Harbin | 055 | MT267428 | MT267465 | MT267369 | MT267399 | AF135835 | MLG32 |
|  |  |  | Harbin | 074 | MT267428 | MT267465 | MT267373 | MT267399 | AF135835 | MLG33 |
|  | MWC_d1 (1) | sika deer (1) | Suihua | 087 | MT267435 | MT267467 | MT267366 | MT267401 | MF496204 | MLG34 |
|  | O (10) | pig (10) | Suihua | 045 | MT267424 | MT267466 | MT267368 | MT267399 | AF267145 | MLG35 |
|  |  |  | Suihua | 053 | MT267424 | MT267466 | MT267368 | MT267399 | AF267145 | MLG35 |
|  |  |  | Suihua | 056 | MT267424 | MT267466 | MT267368 | MT267399 | AF267145 | MLG35 |
|  |  |  | Suihua | 057 | MT267429 | MT267466 | MT267368 | MT267399 | AF267145 | MLG36 |
|  |  |  | Suihua | 058 | MT267430 | MT267466 | MT267368 | MT267399 | AF267145 | MLG37 |
|  |  |  | Suihua | 059 | MT267424 | MT267466 | MT267368 | MT267399 | AF267145 | MLG35 |
|  |  |  | Suihua | 062 | MT267429 | MT267466 | MT267371 | MT267399 | AF267145 | MLG38 |
|  |  |  | Suihua | 070 | MT267429 | MT267466 | MT267368 | MT267399 | AF267145 | MLG36 |
|  |  |  | Suihua | 073 | MT267434 | MT267466 | MT267368 | MT267399 | AF267145 | MLG39 |
|  |  |  | Harbin | 080 | MT267429 | MT267466 | MT267368 | MT267399 | AF267145 | MLG36 |
|  | PigEBITS5 (1) | pig (1) | Harbin | 076 | MT267423 | MT267465 | MT267367 | MT267396 | AF348473 | MLG40 |
| 2 | BEB6 (5) | sheep (5) | Qiqihar | 005 | MT267414 | MT267458 | MT267363 | MT267390 | EU153584 | MLG41 |
|  |  |  | Qiqihar | 022 | MT267415 | MT267459 | MT267364 | MT267389 | EU153584 | MLG42 |
|  |  |  | Suihua | 033 | MT267421 | MT267461 | MT267364 | MT267392 | EU153584 | MLG43 |
|  |  |  | Suihua | 034 | MT267415 | MT267463 | MT267364 | MT267392 | EU153584 | MLG44 |
|  |  |  | Suihua | 035 | MT267415 | MT267458 | MT267364 | MT267392 | EU153584 | MLG45 |
|  | CM7 (2) | sheep (2) | Suihua | 002 | MT267414 | MT267457 | MT267363 | MT267389 | KF543871 | MLG46 |
|  |  |  | Suihua | 032 | MT267414 | MT267462 | MT267363 | MT267390 | KF543871 | MLG47 |
|  | J (1) | dairy cattle (1) | Harbin | 289 | MT267454 | MT267467 | MT267387 | MT267412 | AF135837 | MLG48 |

**Supplementary Table S5 Pairwise genetic distance (*F* _ST_, lower diagonal, *P* < 0.001) and gene flow (*Nm*, upper diagonal) between *E. bieneusi* populations in this study.**

|  | **Subgroup 1** | **Subgroup 2** | **Subgroup 3** |
| --- | --- | --- | --- |
| Subgroup 1 |  | 0.10066 | 0.02977 |
| Subgroup 2 | 0.71294 |  | 0.02205 |
| Subgroup 3 | 0.89358 | 0.91895 |  |

**Supplementary Table S6** **Host ranges of ITS genotypes of *E. bieneusi* used for LD analysis of population genetic structures and sub-structures in the present study^a^.**

| **Cluster** | **Subgroup** | **Phylogenetic group** | **ITS genotype** | **Host** |
| --- | --- | --- | --- | --- |
| 1 | 1 | 1 | CHN-DC1 | cattle, deer, fox, raccoon dog |
|  |  |  | CHN-F1 | fox, raccoon dog |
|  |  |  | CHN-RD1 | deer, rabbit |
|  |  |  | MWC_d1 | deer |
|  |  |  | EbpC | human, beaver, cattle, deer, dog, fox, goat, horse, NHP, muskrat, otter, panda, pig, raccoon, sheep, squirrel, wild boar, bird |
|  |  |  | D | human, bank vole, beaver, cat, cattle, deer, dog, donkey, falcon, fox, goat, hippo, horse, lion, mouse, muskrat, NHP, otter, pig, rabbit, raccoon, raccoon dog, sheep, squirrel, takin, tiger, wild boar, bird |
|  | 2 | 1 | CZ3 | human, horse, mouse |
|  |  |  | H | human, cattle, mouse, pig |
|  |  |  | PigEBITS5 | human, dog, NHP, mouse, pig, wild boar |
|  |  |  | EbpA | human, cattle, deer, dog, goat, horse, mouse, NHP, sheep, pig, wild boar, bird |
|  |  |  | O | human, cattle, dog, horse, NHP, pig, sheep |
|  |  | 2 | J | human, alpaca, bear, cattle, deer, donkey, goat, meerkat, NHP, sheep, yak, zebra, bird |
| 2 | 3 | 2 | BEB6 | human, alpaca, cat, cattle, mouse, deer, goat, horse, NHP, sheep, takin, yak, bird |
|  |  |  | CM7 | cattle, deer, goat, horse, NHP, sheep, yak |

*^a^Information in* ***Supplementary Table S6*** *is obtained based on the work of Li et al. (2019).*

**REFERENCE**

Chen, D., Wang, S. S., Zou, Y., Li, Z., Xie, S.C., Shi, L.Q., et al. (2018). Prevalence and multi-locus genotypes of *Enterocytozoon bieneusi* in black-boned sheep and goats in Yunnan Province, southwestern China. *Infect. Genet. Evol.* 65, 385–391. doi: 10.1016/j.meegid

Deng, L., Li, W., Zhong, Z., Gong, C., Liu, X., Huang, X., et al. (2016a). Molecular characterization and multilocus genotypes of *Enterocytozoon bieneusi* among horses in southwestern China. *Parasit. Vectors* 9:561. doi:10.1186/s13071-016-1844-3

Deng, L., Li, W., Yu, X., Gong, C., Liu, X., Zhong, Z., et al. (2016b). Correction: First Report of the Human-Pathogenic *Enterocytozoon bieneusi* from Red-Bellied Tree Squirrels (*Callosciurus erythraeus*) in Sichuan, China. *PLoS One* 11:e016863. doi: 10.1371/journal.pone.0168631

Deng, L., Li, W., Zhong, Z., Gong, C., Cao, X., Song, Y., et al. (2017). Multi-locus genotypes of *Enterocytozoon bieneusi* in captive Asiatic black bears in southwestern China: High genetic diversity, broad host range, and zoonotic potential. *PLoS One* 12:e0171772. doi: 10.1371/journal.pone.0171772

Desoubeaux, G., Nourrisson, C., Moniot, M., De Kyvon, M. A., Bonnin, V., De La Bretonniére, M. E., et al. (2019). Genotyping Approach for Potential Common Source of *Enterocytozoon bieneusi* Infection in Hematology Unit. *Emerg. Infect. Dis.* 25, 1625–1631. doi: 10.3201/eid2509.190311

Feng, Y., Li, N., Dearen, T., Lobo, M. L., Matos, O., Cama, V., et al. (2011). Development of a multilocus sequence typing tool for high-resolution genotyping of *Enterocytozoon bieneusi*. *Appl. Environ. Microbiol.* 77, 4822–4828. doi: 10.1128/AEM.02803-10

Gui, B. Z., Zou, Y., Chen, Y. W., Li, F., Jin, Y. C., Liu, M. T., et al. (2020). Novel genotypes and multilocus genotypes of *Enterocytozoon bieneusi* in two wild rat species in China: potential for zoonotic transmission. *Parasitol. Res.* 119, 283–290. doi: 10.1007/s00436-019-06491-8

Karim, M. R., Wang, R., He, X., Zhang, L., Li, J., Rume, F. I., et al, (2014). Multilocus sequence typing of *Enterocytozoon bieneusi* in nonhuman primates in China. *Vet. Parasitol.* 200, 13–23. doi: 10.1016/j.vetpar.2013.12.004

Li, W., Cama, V., Feng, Y., Gilman, R. H., Bern, C., Zhang, X., et al. (2012). Population genetic analysis of *Enterocytozoon bieneusi* in humans. *Int. J. Parasitol*. 42, 287–293. doi: 10.1016/j.ijpara.2012.01.003

Li, W., Cama, V., Akinbo, F. O., Ganguly, S., Kiulia, N. M., Zhang, X., et al. (2013). Multilocus sequence typing of *Enterocytozoon bieneusi*: Lack of geographic segregation and existence of genetically isolated sub-populations. *Infect. Genet. Evol.* 14, 111–119. doi: 10.1016/j.meegid.2012.11.021

Li, W., Wan, Q., Yu, Q., Yang,Y., Tao, W., Jiang, Y., et al. (2016a). Genetic variation of mini- and microsatellites and a clonal structure in *Enterocytozoon bieneusi* population in foxes and raccoon dogs and population differentiation of the parasite between fur animals and humans. *Parasitol. Res.* 115, 2899–2904. doi: 10.1007/s00436-016-5069-3

Li, W., Deng, L., Yu, X., Zhong, Z., Wang, Q., Liu, X., et al. (2016b). Multilocus genotypes and broad host-range of *Enterocytozoon bieneusi* in captive wildlife at zoological gardens in China. *Parasit. Vectors* 9:395. doi: 10.1186/s13071-016-1668-1

Li, W., Song, Y., Zhong, Z., Huang, X., Wang, C., Li, C., et al. (2017). Population genetics of *Enterocytozoon bieneusi* in captive giant pandas of China. *Parasit. Vectors* 10:499. doi: 10.1186/s13071-017-2459-z

Li, D., Zheng, S., Zhou, C., Karim, M. R., Wang, L., Wang, H., et al. (2019). Multilocus Typing of *Enterocytozoon bieneusi* in Pig Reveals the High Prevalence, Zoonotic Potential, Host Adaptation and Geographical Segregation in China. *J. Eukaryot. Microbiol.* 66, 707–718. doi: 10.1111/jeu.12715

Luo, R., Xiang, L., Liu, H., Zhong, Z., Liu, L., Deng, L., et al. (2019). First report and multilocus genotyping of *Enterocytozoon bieneusi* from Tibetan pigs in southwestern China. *Parasite* 26:24. doi: 10.1051/parasite/2019021

Ma, Y. T., Zou, Y., Liu, Q., Xie, S. C., Li, R. L., Zhu, X. Q., et al. (2019). Prevalence and multilocus genotypes of *Enterocytozoon bieneusi* in alpacas (*Vicugna pacos*) in Shanxi Province, northern China. *Parasitol. Res.* 118, 3371–3375. doi: 10.1007/s00436-019-06503-7

Song, Y., Li, W., Liu, H., Zhong, Z., Luo, Y., Wei, Y., et al. (2018). First report of *Giardia duodenalis* and *Enterocytozoon bieneusi* in forest musk deer (*Moschus berezovskii*) in China. *Parasit. Vectors* 11:204. doi: 10.1186/s13071-018-2681-3

Tang, C., Cai, M., Wang, L., Guo, Y., Li, N., Feng, Y., et al. (2018). Genetic diversity within dominant *Enterocytozoon bieneusi* genotypes in pre-weaned calves. *Parasit. Vectors* 11:170. doi: 10.1186/s13071-018-2768-x

Tian, G. R., Zhao, G. H., Du, S. Z., Hu, X. F., Wang, H. B., Zhang, L. X., et al. (2015). First report of *Enterocytozoon bieneusi* from giant pandas (*Ailuropoda melanoleuca*) and red pandas (*Ailurus fulgens*) in China. *Infect. Genet. Evol.* 34, 32–35. doi: 10.1016/j.meegid.2015.06.015

Wan, Q., Xiao, L., Zhang, X., Li, Y., Lu, Y., Song, M., et al. (2016). Clonal Evolution of *Enterocytozoon bieneusi* Populations in Swine and Genetic Differentiation in Subpopulations between Isolates from Swine and Humans. *PLoS Negl. Trop. Dis.* 10:e0004966. doi: 10.1371/journal.pntd.0004966

Widmer, G., Dilo, J., Tumwine, J. K., Tzipori, S., and Akiyoshi, D. E. (2013). Frequent occurrence of mixed *Enterocytozoon bieneusi* infections in humans. *Appl. Environ. Microbiol.* 79, 5357–5362. doi: 10.1128/AEM.01260-13

Wu, J., Han, J. Q., Shi, L. Q., Zou, Y., Li, Z., Yang, J. F., et al. (2018). Prevalence, genotypes, and risk factors of *Enterocytozoon bieneusi* in Asiatic black bear (*Ursus thibetanus*) in Yunnan Province, Southwestern China. *Parasitol. Res*. 117, 1139–1145. doi: 10.1007/s00436-018-5791-0

Wu, Y., Chang, Y., Chen, Y., Zhang, X., Li, D., Zheng, S., et al. (2018). Occurrence and molecular characterization of *Cryptosporidium* spp., *Giardia duodenalis*, and *Enterocytozoon bieneusi* from Tibetan sheep in Gansu, China. *Infect. Genet. Evol.* 64, 46–51. doi: 10.1016/j.meegid.2018.06.012

Wang, H. Y., Qi, M., Sun, M. F., Li, D. F., Wang, R. J., Zhang, S. M., et al. (2019). Prevalence and Population Genetics Analysis of *Enterocytozoon bieneusi* in Dairy Cattle in China. *Front. Microbiol.* 10:1399. doi: 10.3389/fmicb.2019.01399

Wang, S. S., Li, J. Q., Li, Y. H., Wang, X. W., Fan, X. C., Liu, X., et al. (2018). Novel genotypes and multilocus genotypes of *Enterocytozoon bieneusi* in pigs in northwestern China: A public health concern. *Infect. Genet. Evol.* 63, 89–94. doi: 10.1016/j.meegid.2018.05.015

Wang, X. T., Wang, R. J., Ren, G. J., Yu, Z. Q., Zhang, L. X., Zhang, S. Y., et al. (2016). Multilocus genotyping of *Giardia duodenalis* and *Enterocytozoon bieneusi* in dairy and native beef (Qinchuan) calves in Shaanxi province, northwestern China. *Parasitol. Res*. 15, 1355–1361. doi: 10.1007/s00436-016-4908-6

Zhang, N., Wu, R., Ji, T., Cui, L. L., Cao, H. X., Li, D., et al. (2020). Molecular Detection, Multilocus Genotyping, and Population Genetics of *Enterocytozoon bieneusi* in Pigs in Southeastern China. *J. Eukaryot. Microbiol.* 67, 107–114. doi: 10.1111/jeu.12759

Zhang, X. X., Cong, W., Lou, Z. L., Ma, J. G., Zheng, W. B., Yao, Q. X., et al. (2016). Prevalence, risk factors and multilocus genotyping of *Enterocytozoon bieneusi* in farmed foxes (*Vulpes lagopus*), Northern China. *Parasit. Vectors* 9:72. doi: 10.1186/s13071-016-1356-1

Zhao, G. H., Du, S. Z., Wang, H. B., Hu, X. F., Deng, M. J., Yu, S. K., et al. (2015). First report of zoonotic *Cryptosporidium* spp., *Giardia intestinalis* and *Enterocytozoon bieneusi* in golden takins (*Budorcas taxicolor bedfordi*). *Infect. Genet. Evol.* 34, 394–401. doi: 10.1016/j.meegid.2015.07.016

Zhong, Z., Li, W., Deng, L., Song, Y., Wu, K., Tian, Y., et al. (2017a). Multilocus genotyping of *Enterocytozoon bieneusi* derived from nonhuman primates in southwest China. *PLoS One* 2:e0176926. doi: 10.1371/journal.pone.0176926

Zhong, Z., Tian, Y., Song, Y., Deng, L., Li, J., Ren, Z., et al. (2017b). Correction: Molecular characterization and multi-locus genotypes of *Enterocytozoon bieneusi* from captive red kangaroos (*Macropus Rfus*) in Jiangsu province, China. *PLoS One* 12:e0190660. doi: 10.1371/journal.pone.0190660

Zhong, Z., Tian, Y., Song, Y., Deng, L., Li, J., Ren, Z., et al. (2017b). Molecular characterization and multi-locus genotypes of *Enterocytozoon bieneusi* from captive red kangaroos (*Macropus Rufus*) in Jiangsu province, China. PLoS One 12:e0183249. doi: 10.1371/journal.pone.0183249.

Zou, Y., Hou, J. L., Li, F. C., Zou, F. C., Lin. R. Q., Ma, J. G., et al. (2018). Prevalence and genotypes of *Enterocytozoon bieneusi* in pigs in southern China. *Infect. Genet. Evol.* 66, 52–56. doi: 10.1016/j.meegid.2018.09.006

Zou, Y., Zheng, W. B., Song, H. Y., Xia, C. Y., Shi, B., Liu, J. Z., et al. (2019). Prevalence and genetic characterization of *Enterocytozoon bieneusi* and *Giardia duodenalis* in Tibetan pigs in Tibet, China. *Infect. Genet. Evol*. 75:104019. doi: 10.1016/j.meegid.2019.104019
